# Isolation and characterization of peroxidase from potato leaves *Solanum tuberosum*: Application in glucose diagnostic kit

**DOI:** 10.1016/j.jgeb.2025.100584

**Published:** 2025-09-30

**Authors:** Hassan M.M. Masoud, Mohammed M. Abdel-Monsef, Mohamed S. Helmy, Sayed S. Esa, Doaa A. Darwish

**Affiliations:** a Molecular Biology Department, National Research Centre, El-Tahrir St., Dokki, Giza P.O. 12622, Egypt; b Proteome Research Laboratory, National Research Centre, El-Tahrir St., Dokki, Giza P.O. 12622, Egypt

**Keywords:** Potato Leaves, Peroxidase, Purification, Characterization, Glucose diagnostic kit

## Abstract

Peroxidases play a pivotal role in many medical applications such as diagnostic kits and ELISA assays. This study reports the purification and biochemical characterization of peroxidase from potato leaves (PLPOD) and its application in the formulation of a glucose diagnostic kit. PLPOD was purified through CM-cellulose ion-exchange and Sephacryl S-300 gel filtration chromatography, achieving an 11.8-fold purification with 48 % recovery and a final specific activity of 705.7 U/mg. Native PAGE and activity staining confirmed the enzyme’s purity and homogeneity. PLPOD molecular weight was estimated from gel filtration column as 64 kDa, but on SDS gel, there were three PLPOD isoforms of approximated molecular weights ranging from ∼ 40–60 kDa. PLPOD exhibited optimal activity at pH 5.2, with Zn^2+^ and Ni^2+^ enhancing activity, while Ca^2+^ and Fe^2+^ inhibited it. Inhibitor analysis confirmed the heme-dependent nature of the enzyme. The *K_m_* values for guaiacol and H_2_O_2_ were 0.067 mM and 40 mM, respectively, consistent with typical plant peroxidases. A glucose diagnostic kit developed using PLPOD showed strong concordance with a commercial glucose kit when tested on normal and diabetic serum samples demonstrating its clinical applicability. These findings suggest that PLPOD is a viable cost-effective for use in diagnostic assays and kits.

## Highlights

• Potato leaves peroxidase (PLPOD) was purified through two chromatographic steps.
• PLPOD achieved 11.8-fold purification times with a specific activity of 705.7 U/mg.
• PLPOD molecular weight was ranging from ∼ 40–60 kDa.
• PLPOD exhibited optimal activity at pH 5.2, with Zn^2+^ and Ni^2+^ enhancing activity.
• PLPOD was applied in constructing a glucose diagnostic kit.

## Graphical abstract

**Figure:**
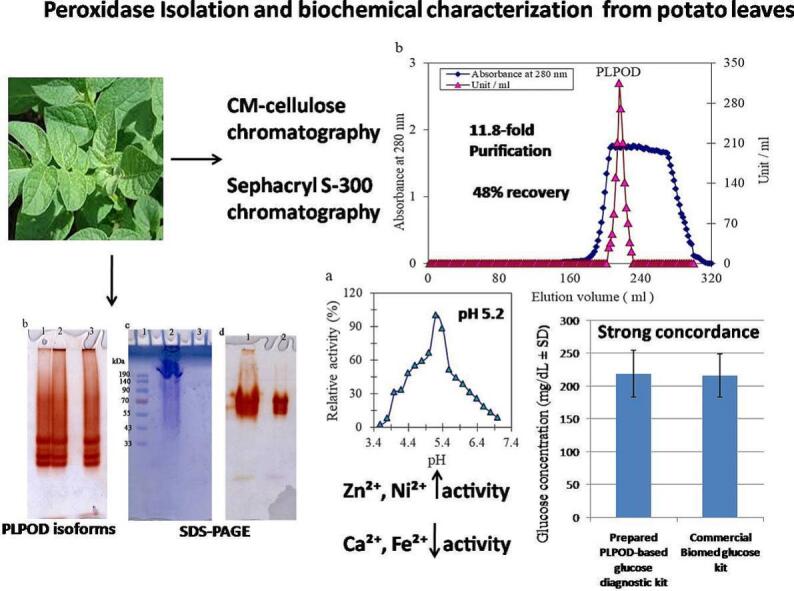

## 1 Introduction

Peroxidases (EC 1.11.1.x) are heme-containing enzymes that catalyze the reduction of hydrogen peroxide (H_2_O_2_) using a variety of electron-donating substrates.^1^ These enzymes are widely distributed in nature and play essential roles in diverse biological functions such as lignification, suberization, auxin catabolism, defense against pathogens, and oxidative stress responses.^2^, ^3^

In plants, peroxidase is especially important and are involved in cell wall stiffening, reactive oxygen species (ROS) scavenging, and programmed cell death.^4^, ^5^ Numerous plant species have been investigated for peroxidase isolation, including sweet potato (Ipomoea batatas), tomato (Solanum lycopersicum), soybean (Glycine max) and radish (Raphanus sativus).^6^, ^7^ *Solanum tuberosum* (potato) is not only one of the world’s most important staple crops, but also a potential bioresource for enzymes due to the presence of peroxidase in its tissues.^8^ Previous studies have shown that potato tubers and leaves contain measurable levels of peroxidase activity.^9^ The activity of peroxidase in potato leaves is influenced by developmental stage and environmental stress, making it a useful biochemical marker for plant physiology studies.^10^

Beyond their physiological importance, plant peroxidases have drawn attention for their practical applications in biotechnology. Due to their ability to catalyze chromogenic reactions, they are widely used in immunoassays, biosensors, and diagnostic kits.^11^ Horseradish peroxidase (HRP) remains the most commercially used, challenges such as high production costs and limited stability drive the search for alternative sources.^12^ Leaf peroxidases from crops like *Ipomoea batatas* and *Raphanus sativus* have shown promising enzymatic properties and stability.^6^, ^13^ Peroxidase is widely utilized as reporter molecules in various biochemical assays such as enzyme-linked immunosorbent assays (ELISA), immunohistochemistry (IHC), western blotting, and biosensor technologies^14^, ^15^ and allowing for the quantitative or qualitative detection of a wide range of analytes, including hormones, antibodies, antigens, pathogens, and drugs.^16^, ^17^

In clinical laboratory, one of the most established and sensitive methods for colorimetric glucose determination involves a system composed of glucose oxidase (GOx), peroxidase, 4-aminoantipyrine (4-AAP) and in combination with a phenol. In this system GOx catalyzes the oxidation of glucose to gluconic acid producing hydrogen peroxide (H_2_O_2_) as a byproduct. Then peroxidase uses the H_2_O_2_ to oxidize 4-AAP and a phenol generating a quinoneimine dye which has a distinct pink-red color with an absorbance maximum typically around 505–510 nm.^18^, ^19^ This dye intensity is directly proportional to glucose concentration and is easily measurable by a spectrophotometer making the system ideal for clinical biochemistry laboratories and automated analyzers.^20^ This assay is especially valued for its high sensitivity, specificity, and stability, and is widely used in serum, plasma, cerebrospinal fluid, and even food analysis.^21^, ^22^ Peroxidase enzyme is crucial in this cascade as it enables the chromogenic reaction by catalyzing the coupling between 4-AAP and phenol in the presence of H_2_O_2_. However, horseradish peroxidase (HRP) may suffer from thermal and pH instability, prompting interest in alternative sources like plant-derived peroxidases (e.g., from *Solanum tuberosum* or *Raphanus sativus*) which offer superior storage and operational stability.^23^, ^24^

Despite the economic importance of potato, limited research has been conducted on the isolation and detailed biochemical characterization of peroxidase from its leaves. Since potato leaves are a readily available agricultural by-product, valorizing them through enzyme extraction can be both economically and environmentally beneficial. This study focuses on the isolation and characterization of peroxidase from *Solanum tuberosum* leaves, with an aim to assess its enzymatic properties and potential application in construction of glucose diagnostic kit for future industrial and diagnostic technologies.

## 2 Materials and methods

### 2.1 Sample

Fresh leaves of Solanum tuberosum (potato) were collected from cultivated fields in Egypt during the active vegetative growth stage. Samples were obtained from healthy, disease-free plants grown under standard agronomic practices in the Nile Delta region. The leaves were harvested in the early morning. Fully expanded mature leaves were selected. Immediately after detachment, the leaf samples were rinsed with distilled water to remove dust and surface contaminants, blotted dry, and kept in sterile bags, the samples were stored on ice and transported promptly to the laboratory. Approximately 50 g of leaf tissue were ground in a pre-chilled mortar and pestle using 100 mL of ice-cold 50 mM phosphate buffer (pH 7.5). The homogenate was centrifuged at 12,000 rpm for 15 min at 4 °C to remove cellular debris. The resulting clear supernatant was collected as the crude peroxidase extract and stored at –20 °C during further analysis.

### 2.2 Chemicals

Guaiacol, CM-cellulose, DTT (dithiothreitol), 1,10 phenanthroline, bovine serum albumin (BSA), 4-Aminoantipyrine, glucose oxidase (GOx), phenol, Sephacryl S-300 and kits of gel filtration molecular weight marker were product of Sigma Co. SDS molecular weight marker proteins were purchased from Pharmacia Co. Other chemicals were of analytical grade.

### 2.3 Peroxidase (POD) activity assay

Peroxidase activity was determined using the guaiacol oxidation method.^25^ The reaction mixture consisted of 8μ moles of H_2_O_2_, 60 μmoles guaiacol and 150 μmoles sodium acetate buffer at pH 5.6. The enzyme activity was determined by measuring the difference in absorbance (0.01) at 470 nm for 3 min.

### 2.4 Peroxidase activity staining on PAGE

After electrophoresis, the gel was dipped in a freshly formulated solution containing 266 μmoles of H_2_O_2_ and 2000 μmoles of guaiacol in 100 ml of 0.05 M sodium acetate buffer, pH 5.6, with 7 % acetic acid blocking the enzymatic reaction.^26^

### 2.5 Purification of potato leaves peroxidase (PLPOD)

#### 2.5.1 Chromatography on CM-cellulose column

The purification of peroxidase from potato leaves (PLPOD) was performed at 4 °C to preserve enzyme stability and activity throughout the procedure. The crude extract was first subjected to ion-exchange chromatography on a CM-cellulose column (2.4 × 12 cm) previously equilibrated with 20 mM sodium acetate buffer (pH 5.0). The sample was loaded onto the column and proteins were eluted using a stepwise NaCl gradient in the same buffer at a flow rate of 60 mL/h. Fractions of 5 mL were collected, and peroxidase activity and protein concentration were determined. Fractions exhibiting high specific peroxidase activity were pooled and concentrated by lyophilization for further purification.

#### 2.5.2 Chromatography on column Sephacryl S-300

The concentrated active protein pool was then applied to a Sephacryl S-300 gel filtration column (1.75 × 142 cm), pre-equilibrated with 20 mM sodium acetate buffer (pH 5.0). The chromatography was run at a flow rate of 30 mL/h, with 2 mL fractions collected for analysis. The column was calibrated using molecular weight standards ranging from 17 to 440 kDa to estimate the native molecular mass of PLPOD. Throughout all purification steps, enzyme activity was monitored and protein content was quantified ensuring assessment of specific activity improvements at each stage.

### 2.6 Electrophoretic analysis

Gel electrophoresis was achieved using 7 % PAGE.^27^ SDS-PAGE was carried out with 12 % PAGE.^28^ The molecular weight of purified peroxidase (PLPOD) was determined using SDS-PAGE.^29^ Proteins stained using Coomassie brilliant blue (R-250) 0.25 % conc.

### 2.7 Protein determination

Protein concentrations were determined during the purification steps spectrophotometrically according to Bradford method.^30^

### 2.8 Effect of pH

The optimum pH for activity of purified PLPOD was carried out utilizing buffer 20 mmol L^-1^ (Na-acetate buffer, pH 3.6 to 5.6, Na-phosphate buffer, pH 5.7 to 7.0).

### 2.9 Effect of cations

The divalent cations effect on activity of purified PLPOD was measured after 5 mM for each cation pre-incubation at 37˚C. A without cation control is taken 100 % activity.

### 2.10 Effect of inhibitors

The effect of inhibitors on activity of purified PLPOD was measured after 5 mM pre-incubation for each inhibitor at 37˚C. A control without inhibitor is taken 100 % activity.

### 2.11 Michaelis-Menten constant (*K_m_*)

The purified PLPOD was incubated with increasing concentrations of guaiacol and H_2_O_2_. A definite amount of PLPOD was used to construct the plot for the reciprocal of reaction velocity (V) versus the substrate concentration [S].

### 2.12 Preparation of glucose diagnostic kit

The glucose diagnostic kit is based on an enzymatic colorimetric assay that utilizes glucose oxidase (GOx) and peroxidase (POD). In this kit, glucose is first oxidized by GOx to produce gluconic acid and hydrogen peroxide (H_2_O_2_). The generated H_2_O_2_ is then reduced by POD using a chromogenic substrate (4-aminoantipyrine with phenol), producing a colored product measurable at 510–520 nm. The glucose diagnostic kit was prepared using an enzymatic colorimetric method based on the coupled action of GOx and peroxidase (PLPOD) that purified from potato leaves. The working reagent was formulated in a 100 mM phosphate buffer at pH 7.0, which provides optimal activity for both enzymes. The solution contained glucose oxidase at 15,000 U/L (equivalent to 1500 U in 100 mL) to catalyze the oxidation of glucose to gluconic acid and hydrogen peroxide. Peroxidase was added at a concentration of 1000 U/L (100 U per 100 mL), to facilitate the subsequent reaction with hydrogen peroxide. For the chromogenic system, 4-aminoantipyrine (0.5 mM) and phenol (10 mM) were included; these react in the presence of hydrogen peroxide and POD to form a pink quinoneimine dye, measurable at 510–520 nm. The glucose standards for calibration were prepared from a 1 g/L stock solution of D-glucose in distilled water, with working standards ranging from 0 to 200 mg/dL. During the assay, 1.0 mL of the working reagent was mixed with 10 µL of the sample or standard and incubated at 37 °C for 10 min. The absorbance was measured at 510 nm against a reagent blank. The intensity of the developed color was directly proportional to the glucose concentration. The concentration of glucose in the sample was calculated by comparing the absorbance of the test sample (A-sample) to that of a standard glucose solution (A-standard) of (a standard glucose solution of 100 mg/dL).^31^, ^32^

### 2.13 Statistical analysis

All experiments were performed in triplicate, and the results are expressed as the mean ± standard deviation (SD). Glucose concentrations measured were statistically compared using Minitab software (Version 22.1.0). Paired Student’s *t*-test was performed to evaluate whether there was a statistically significant difference in mean glucose levels obtained from both kits. A p-value ≤ 0.05 was considered statistically significant. Pearson correlation coefficient (r) was calculated to assess the strength and direction of the linear association between the two kits.^33^, ^34^

## 3 Results

### 3.1 Purification of potato leaves peroxidase (PLPOD)

The crude extract contained 120 mg of total protein with 7200 enzyme units, resulting in a specific activity of 60.0 U/mg protein. Following CM-cellulose ion-exchange chromatography, the protein content was reduced to 52.4 mg, with a total activity of 4805 U, yielding a specific activity of 91.7 U/mg. This step recovered 66.7 % of the initial activity and achieved a 1.5-fold purification (Table 1). The final purification step using Sephacryl S-300 gel filtration chromatography resulted in a significant enhancement of purity. Only 4.9 mg of protein was obtained retaining 3458 U of enzymatic activity corresponding to a specific activity of 705.7 U/mg. This final step provided a 48.0 % yield with an overall 11.8-fold purification compared to the crude extract (Table 1). The molecular weight of PLPOD was determined from gel filtration column elution volume to be 64 kDa.

**Table 1:** A typical purification scheme of potato leaves POD isoenzymes.

Purification step	Total mg proteins	Total units	Recovery (%)	Specific activity	Fold purification
Potato leaves crude extract	120	7200	100.0	60.0	1.0
CM-cellulose fraction	52.4	4805	66.7	91.7	1.5
Sephacryl S-300 fraction	4.9	3458	48.0	705.7	11.8

### 3.2 Electrophoretic analysis

**Fig. 1:**
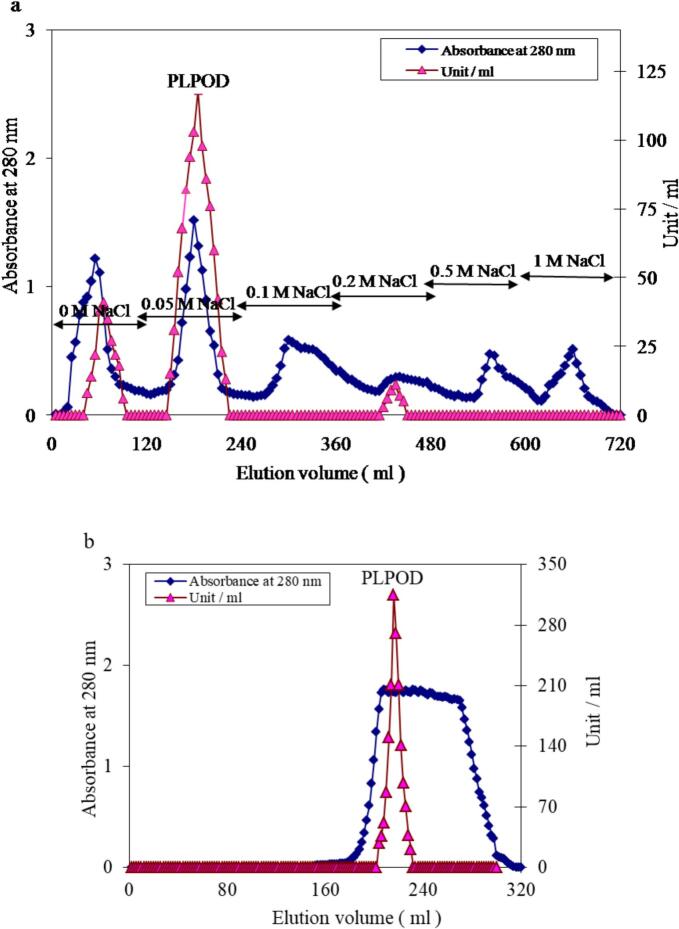
(a) Elution profile of potato leaves crude extract on a CM-cellulose column (12 × 2.4 cm), pre-equilibrated with 20 mM sodium acetate buffer (pH 5.0). (b) A typical elution profile of the concentrated PLPOD-active pooled fractions from CM-cellulose on a Sephacryl S-300 column (142 × 1.75 cm) pre-equilibrated with 20 mM sodium acetate buffer (pH 5.0).

**Fig. 2:**
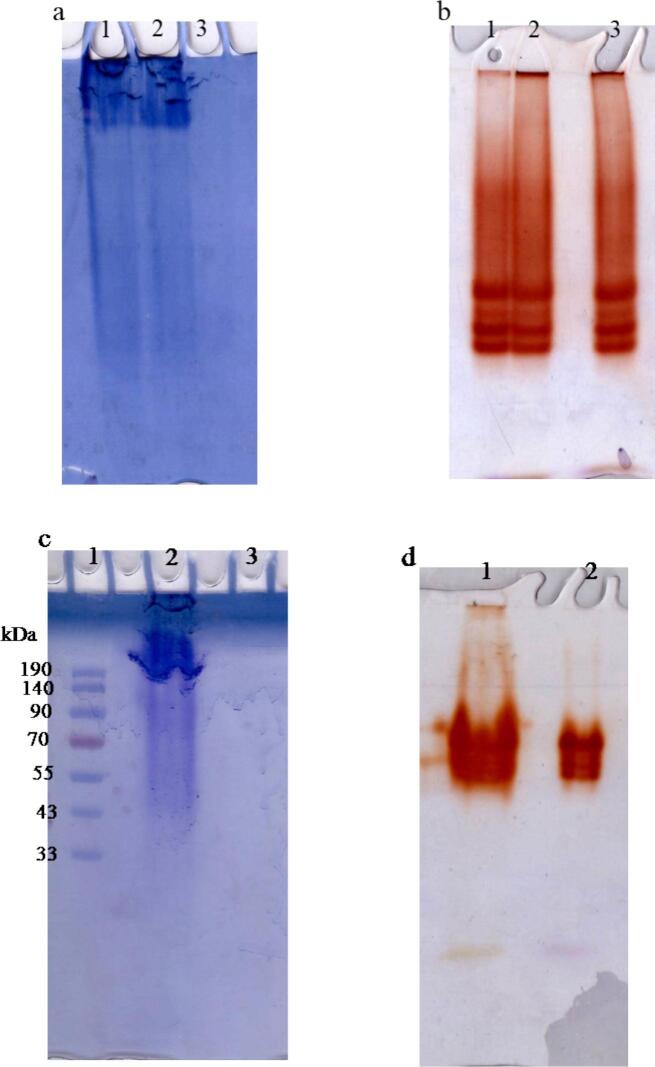
(a) Electrophoretic analysis of PLPOD protein pattern on 7% native PAGE; (1) crude extract, (2) CM-cellulose fraction and (3) Sephacryl S-300 fraction. (b) PLPOD isoenzyme pattern on 7% native PAGE: (1) crude extract, (2) CM-cellulose fraction, (3) Sephacryl S-300 fraction. (c) Electrophoretic analysis of PLPOD protein pattern on 12% SDS-PAGE: (1) Molecular weight marker proteins, (2) crude extract, (3) denatured purified PLPOD, (d) PLPOD isoenzyme pattern on 12% SDS-PAGE: (1) crude extract, (2) purified PLPOD.

The homogeneity of the purified potato leaf peroxidase (PLPOD) was evaluated using native 7 % polyacrylamide gel electrophoresis (PAGE). Samples from each purification step including the crude extract, CM-cellulose fraction, and Sephacryl S-300 fraction were loaded and compared. Lanes 1 and 2 (crude extract and CM-cellulose fraction) in protein Gel (Fig. 2a) showed smeared and dense multiple bands indicating a complex protein mixture, while in POD activity Gel (Fig. 2b) there are ∼ 4 distinct POD isoenzyme bands visible confirming that potato leaves express multiple POD isoforms. Lane 3 (purified PLPOD) in protein Gel showed one or two faint protein bands visible — looks quite pure, while in activity Gel there are 3 notable POD isoenzyme bands remain, sharper and more intense potentially corresponds to the single band seen in the protein gel. On SDS PAGE (Fig. 2c), lane 2 contains a thick blue-stained band near the top of the gel, with some smearing faint bands downward suggesting a highly abundant protein, and the smearing below may indicate partial degradation or multiple isoforms of the enzyme. Lane 3 (denatured purified PLPOD) appeared to be mostly blank. On visualizing the purified PLPOD activity on SDS PAGE (Fig. 2d), Lane 1 (crude leaves extract) showed multiple distinct reddish-brown bands at various molecular weights. Lane 2 (native purified PLPOD) showed fewer and lighter bands Indicating reduced POD isoenzyme expression may be due to treatment effect. The molecular weight of the PLPOD isoenzymes can be approximately estimated by correlating their migration distances with the protein ladder in Fig. 1c. The top POD band is ∼ 60–65 kDa, the middle band is ∼ 45–50 kDa, and the faint lower band is ∼ 40–45 kDa.

### 3.3 Effect of pH

**Fig. 3:**
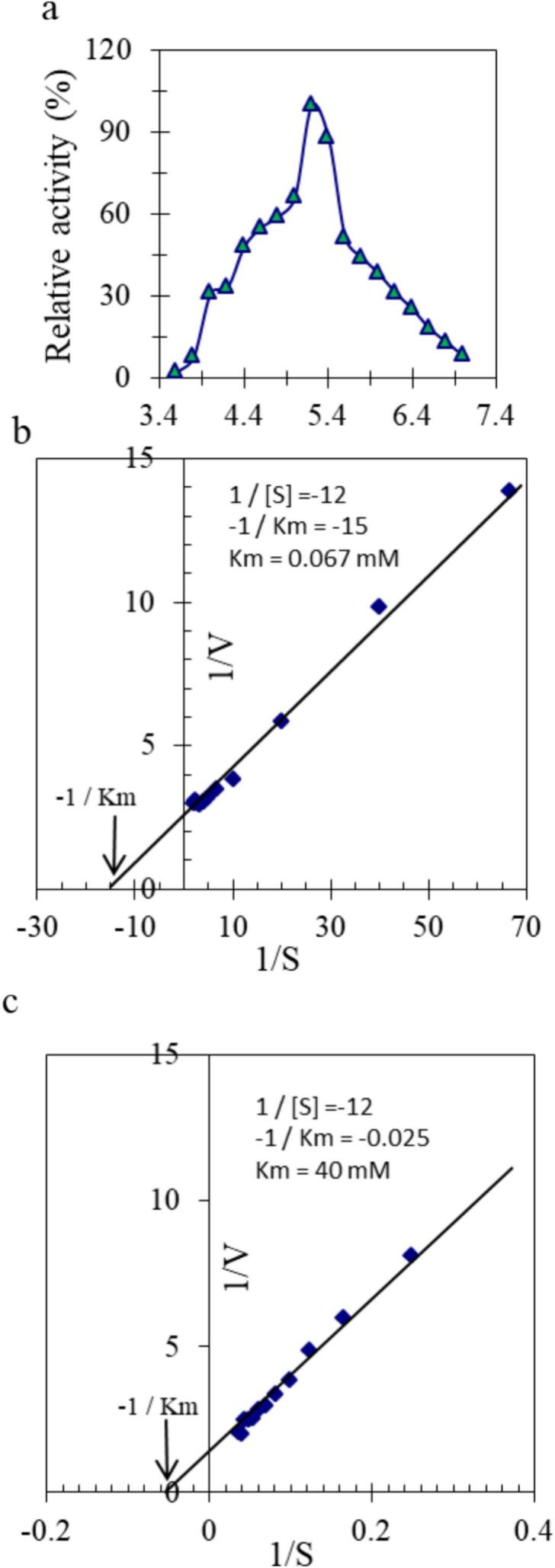
The optimum pH for activity of purified PLPOD was carried out utilizing buffer 20 mmol L-1 pH 3.4 to 7.0 (Na-acetate and Na-phosphate). (a) Lineweaver-Burk plot relating the reciprocal of the reaction velocity of the purified PLPOD to Guaiacol concentration in mM. (b) Lineweaver-Burk plot relating the reciprocal of the reaction velocity of the purified PLPOD to H_2_O_2_ concentration in mM.

The effect of pH on the activity of purified potato leaf peroxidase (PLPOD) was evaluated using a 20 mmol L^−1^ buffer system comprising Na-acetate buffer (pH 3.6–5.6) and Na-phosphate buffer (pH 5.7–7.0). The enzyme exhibited varying degrees of activity across the tested pH range, with a distinct maximum at pH 5.2 (Fig. 3a).

### 3.4 Michaelis-Menten constant (*K_m_*)

The PLPOD obeyed the Michaelis-Menten kinetics and a linear relationship between (V) and [S] was obtained ended with a steady state at the maximum velocity. A Lineweaver-Burk plot for the reciprocal of the reaction velocity (1/V) and substrate concentration (1/[S]) was constructed, the *K_m_* value of PLPOD was deduced to be 0.067 mM for guaiacol (Fig. 3b) and 40 mM for H_2_O_2_ (Fig. 3c).

### 3.5 Effect of cations

The influence of various divalent metal ions (5 mM) on the activity of purified potato leaf peroxidase (PLPOD) was investigated, and the results are summarized in (Table 2). Among the tested cations, Zn^2+^ and Ni^2+^ significantly enhanced PLPOD activity, with relative activities of 115.7 % and 110.4 % respectively, compared to the control. Mn^2+^, Co^2+^, Mg^2+^, and Cu^2+^ caused moderate inhibition, retaining 81.0 %, 78.6 %, 72.3 %, and 71.3 % activity respectively. Fe^2+^ exhibited a stronger inhibitory effect (61.7 % relative activity), while Ca^2+^ caused the most pronounced inhibition, reducing activity to 34.4 %.

**Table 2:** Effect of divalent cations on the purified potato leaves PLPOD.

Reagent (5 mM)	Residual activity (%)
Control	100.0
CaCl_2_	34.4 ± 2.17
CoCl_2_	78.6 ± 3.12
CuCl_2_	71.3 ± 1.62
FeCl_2_	61.7 ± 2.38
MgCl_2_	72.3 ± 2.08
MnCl_2_	81.0 ± 1.88
NiCl_2_	110.4 ± 2.98
ZnCl_2_	115.7 ± 4.48

* These values represent % of the control and the means of triplicate experiments ± SD.

### 3.6 Effect of inhibitors

The sensitivity of purified PLPOD to various inhibitors was assessed at a concentration of 5 mM and the percentage inhibition relative to the control is presented in (Table 3). Among the tested inhibitors, potassium cyanide (KCN) exhibited the strongest inhibition with 99.6 % activity loss, followed closely by sodium azide (NaN_3_, 95.2 %) and DTT (93.4 %), indicating potent interference with enzymatic activity. β-mercaptoethanol also caused substantial inhibition (82.6 %), while N-ethylmaleimide and PMSF showed moderate effects with 35.6 % and 27.6 % inhibition, respectively. Sodium dodecyl sulfate (SDS) reduced activity by 14.9 %, whereas 1,10-phenanthroline resulted in 29.3 % inhibition. Sodium dichromate and EDTA exhibited no inhibitory effect maintaining 100 % residual activity.

**Table 3:** Effect of inhibitors on the purified PLPOD.

Inhibitor (5 mM)	Inhibition %
Control	0.0
Potassium cyanide (KCN)	99.6 ± 2.62
N-ethylmlimide	35.6 ± 3.14
Sodium Azide (NaN_3)_	95.2 ± 3.43
Sodium dodecyl sulphate (SDS)	14.9 ± 1.52
DL-Dithiothreitol (DTT)	93.4 ± 4.19
β-Mercaptoethanol	82.6 ± 2.68
Sodium dichromate	0.0
1,10-Phenanethroline	29.3 ± 3.76
EDTA	0.0
PMSF	27.6 ± 3.29

* These values represent % of the control and the means of triplicate experiments ± SD.

### 3.7 Preparation of glucose diagnostic kit

A glucose diagnostic kit was developed using the purified potato leaf peroxidase (PLPOD) and tested for its efficacy on serum samples from 30 individuals, including normal (Samples 1–6) and diabetic patients (Samples 7–30). The glucose concentrations measured by the prepared kit were compared with those from a commercial Biomed glucose diagnostic kit. Results showed a strong agreement between the two kits across all sample types—FBS (fasting), PBS (postprandial), and RBS (random blood sugar) (Table 4). The constructed PLPOD-based glucose diagnostic kit showed excellent agreement with the commercial Biomed kit, with no significant difference in paired *t*-test results (p = 0.378). The strong correlation (r = 0.993, p < 0.001) confirmed its reliability across normal and diabetic samples.

**Table 4:** Comparing of the constructed kit (using PLPOD) with commercially available Biomed glucose diagnostic kit using diabetic patient samples.

Serum samples		Prepared glucose kit (mg/dL ± S.D)	Commercial kit (mg/dL ± S.D)	Paired *t*-test (p-value)	Correlation coefficient (r)
1	RBS	94.10 ± 1.63	97.64 ± 1.89	p = 0.378 (No significant difference)	r = 0.993 → Strong positive correlation
2	FBS	68.51 ± 1.76	88.26 ± 1.14		
3	PBS	116.57 ± 3.99	112.25 ± 3.30		
4	FBS	89.75 ± 1.85	88.35 ± 2.97		
5	PBS	96.31 ± 1.98	91.46 ± 2.03		
6	RBS	57.64 ± 1.87	68.62 ± 1.44		
7	FBS	140.46 ± 1.56	146.36 ± 1.73		
8	PBS	196.11 ± 2.15	206.31 ± 2.87		
9	FBS	159.61 ± 1.86	163.03 ± 2.04		
10	PBS	366.54 ± 3.56	344.01 ± 3.95		
11	FBS	108.34 ± 0.89	115.66 ± 0.72		
12	PBS	170.10 ± 1.26	183.28 ± 1.08		
13	FBS	237.41 ± 2.54	200.24 ± 1.79		
14	PBS	402.92 ± 4.87	423.37 ± 3.95		
15	FBS	113.56 ± 1.32	104.27 ± 1.88		
16	PBS	271.64 ± 3.37	248.56 ± 2.99		
17	FBS	334.80 ± 4.27	348.58 ± 3.65		
18	PBS	408.67 ± 3.55	392.05 ± 4.12		
19	FBS	103.19 ± 0.89	95.91 ± 0.47		
20	PBS	239.92 ± 1.73	227.40 ± 1.46		
21	FBS	395.31 ± 2.88	371.74 ± 3.57		
22	PBS	496.37 ± 4.69	465.82 ± 5.18		
23	FBS	269.51 ± 3.43	254.23 ± 2.99		
24	PBS	395.16 ± 5.84	389.28 ± 6.32		
25	FBS	135.60 ± 1.18	155.27 ± 1.57		
26	PBS	207.91 ± 2.07	219.86 ± 1.98		
27	FBS	121.64 ± 1.11	110.13 ± 1.59		
28	PBS	279.97 ± 6.15	290.14 ± 4.76		
29	FBS	170.10 ± 4.83	183.28 ± 3.57		
30	PBS	324.73 ± 7.46	309.41 ± 5.82		

Data are shown as mean ± SD, FBS: Fasting Blood Sugar; PBS: Postprandial Blood Sugar; RBS: Random Blood Sugar; Sample 1–6 (normal individuals); Sample 7–30 (diabetic individuals), * significant P-value (p ≤ 0.05), Correlation coefficient (0.8 ≤ r ≤ 1.0 → very high correlation).

## 4 Discussion

Peroxidases play a pivotal role in medical applications such as the diagnostic kits and ELISA due to their strong oxidative characteristics and substrate specificity.^35^ Here, the purification results of peroxidase enzyme (PLPOD) from potato leaves demonstrated the effectiveness of the two-step chromatography protocol in significantly enhancing enzyme purity while maintaining reasonable activity yields. The initial crude extract displayed a low specific activity (60.0 U/mg), as expected due to the presence of various non-enzymatic proteins and plant debris. Following CM-cellulose ion-exchange chromatography, a modest increase in specific activity to 91.7 U/mg and a recovery of 66.7 % was achieved, indicating partial removal of contaminants and enrichment of peroxidase proteins. The most substantial purification occurred after gel filtration chromatography using Sephacryl S-300, where an increase in specific activity to 705.7 U/mg was observed, corresponding to an 11.8-fold purification and a recovery of 48.0 %. The overall purification yield was consistent with those reported for other plant-derived peroxidases such as from radish, onion, or turnip.^36^, ^37^ The significant increase in specific activity reflects successful isolation of active peroxidase isoform. The results also suggest that potato leaves are a viable, cost-effective source of peroxidase with potential for biotechnological applications, such as in diagnostic kits, particularly where plant-based enzymes are desirable alternatives to horseradish peroxidase.^38^, ^39^

The electrophoretic profile of the purified peroxidase from potato leaves (PLPOD) was evaluated using native 7 % PAGE to assess the purity and homogeneity of the enzyme following each purification step. The crude extract and CM-cellulose fraction in protein Gel (Fig. 2a) showed smeared and dense multiple bands indicating a complex protein mixture, while in POD activity Gel (Fig. 2b) showed ∼ 4 distinct POD isoenzyme bands visible confirming that potato leaves express multiple POD isoforms. The purified PLPOD in protein Gel showed one or two faint protein bands visible — looks quite pure, while in activity Gel there are 3 notable POD isoenzyme bands remain, sharper and more intense potentially corresponds to the single band seen in the protein gel. These findings confirm the successful purification of PLPOD active form., however, possibly some isoforms were lost during purification — maybe due to differential solubility, pH sensitivity, or affinity behavior. On SDS PAGE (Fig. 2c), denatured purified PLPOD didn’t appear on the gel, but on visualizing the purified PLPOD activity on SDS PAGE (Fig. 2d), 2–3 reddish-brown POD activity bands were seen. PLPOD isoenzymes have approximate molecular weights of about 60–65 kDa, 45–50 kDa, and 40–45 kDa in correlation of their migration distances with the protein ladder, which aligns with its molecular weight determined from the gel filtration column and with most known peroxidase isoforms in plants that commonly range between 40–60 kDa.^40^, ^41^, ^42^ PLPOD molecular mass estimated by gel filtration (64 kDa) was slightly differing from that observed on SDS-PAGE (40–60 kDa). Such variations may arise due to glycosylation and the hydration shell of the protein, which affect native-state estimations, whereas SDS-PAGE separates the denatured isoforms of the polypeptide. The presence of multiple bands in SDS-PAGE further suggests that PLPOD exists in different isoforms, as reported for horseradish peroxidases.^43^, ^44^

The pH profile of the purified PLPOD revealed a clear maximum in enzymatic activity at pH 5.2, indicating that the enzyme functions optimally under mildly acidic conditions. This is consistent with the behavior of many plant-derived peroxidases, such as those from radish, soybean, and horseradish, which commonly show optimal activity between pH 4.5 and 6.0.^36^, ^45^ The effect of divalent cations on PLPOD activity showed a wide range of influences, highlighting the enzyme’s sensitivity to metal ion interactions. The strong enhancement by Zn^2+^ and Ni^2+^ suggests that these ions may either stabilize the enzyme conformation or positively interact with the heme environment enhancing electron transfer during catalysis. Similar stimulatory effects of Zn^2+^ have been reported in peroxidases from other plant sources such as soybean and radish.^36^, ^46^ In contrast, the marked inhibition by Ca^2+^ (34.4 % residual activity) indicates a potential destabilizing or blocking effect on the enzyme’s active site which is also observed in horseradish and onion peroxidases.^45^ There is a moderate inhibition by Fe^2+^, Cu^2+^, and Co^2+^. The mild inhibition by Mn^2+^ and Mg^2+^ suggests these ions do not significantly alter the enzyme’s conformation. The inhibition profile of purified PLPOD revealed critical insights into the enzyme's catalytic mechanism and sensitivity to chemical agents. The nearly complete inhibition by KCN and NaN_3_ confirms the presence of a heme prosthetic group at the active site, as both are well-known ligands that bind strongly to the iron center of heme-containing enzymes, disrupting electron transfer and catalysis.^47^, ^48^ Similarly, the strong inhibitory effects of DTT and β-mercaptoethanol suggest the involvement of disulfide bonds in maintaining the enzyme’s tertiary structure, which are reduced by these agents, leading to conformational destabilization and loss of activity.^36^ The partial inhibition by N-ethylmaleimide, a thiol-reactive compound, supports the possible involvement of cysteine residues in catalytic or structural roles. In contrast, the minimal inhibition by EDTA and sodium dichromate indicates these agents do not disrupt the active site architecture. The moderate effects of SDS and PMSF may reflect mild denaturation or interference with essential serine residues, though serine does not appear to be critical to catalysis. Additionally, 1,10-phenanthroline, a metalloprotease inhibitor, had a mild impact, reinforcing that metal chelation does not significantly affect enzyme function. The kinetic behavior of the purified potato leaf peroxidase (PLPOD) followed classical Michaelis-Menten kinetics indicating a single-substrate catalytic mechanism with saturation behavior at higher substrate concentrations. The Lineweaver-Burk plots yielded *K_m_* values of 0.066 mM for guaiacol and 40 mM for H_2_O_2_ suggesting a higher affinity for guaiacol as the electron donor compared to hydrogen peroxide as the oxidizing substrate. These values are consistent with those reported for other plant peroxidases such as horseradish (*K_m_* for guaiacol ≈ 0.03–0.1 mM) and radish peroxidase (*K_m_* for H_2_O_2_ ≈ 25–50 mM), supporting the current results.^45^, ^46^ The low *K_m_* for guaiacol reflects efficient substrate binding which is desirable for colorimetric assays and diagnostic applications where guaiacol is commonly used as a chromogenic substrate. In contrast, the relatively higher *K_m_* for H_2_O_2_ implies that higher concentrations of the oxidant are needed to achieve maximal enzyme velocity which is a typical characteristic of many plant-derived peroxidases.^36^, ^49^ These kinetic parameters indicate that PLPOD possesses robust catalytic efficiency and is potentially suitable for use in diagnostic kits targeting oxidative reactions particularly for glucose monitoring systems that rely on hydrogen peroxide detection. The prepared glucose diagnostic kit employing purified PLPOD demonstrated high accuracy and consistency when benchmarked against the commercial Biomed kit. A paired Student’s *t*-test showed no statistically significant difference between the values obtained by the two kits (p = 0.378), which indicates that the prepared diagnostic kit produced results comparable to those of the commercial kit. To further evaluate agreement, Pearson correlation analysis was performed. A very strong positive correlation was found between the prepared and commercial kits (r = 0.993, p ≤ 0.05), which suggests that the two kits provide highly consistent measurements. The strong correlation in glucose concentration readings across fasting, postprandial, and random samples supports the enzyme’s reliability in catalyzing the chromogenic reaction with hydrogen peroxide generated from glucose oxidation, consistent with the glucose oxidase–peroxidase (GOD-POD) assay principle.^36^, ^48^, ^50^, ^51^ Among normal individuals, glucose values determined by the prepared kit ranged from 57.64 to 116.57 mg/dL, compared to 68.62 to 112.25 mg/dL using the commercial kit. For diabetic patients, glucose values with the prepared kit ranged between 89.75 and 496.37 mg/dL, closely matching those of the commercial kit (88.35 to 465.82 mg/dL). Slight variations were observed between individual sample results but the overall trends and diagnostic capability remained consistent confirming the practical utility of the prepared kit. Minor variations in values between the kits, yet all measurements fell within diagnostically acceptable ranges. Nevertheless, the practical performance especially in diabetic samples with a wide glucose range (89.75 to 496.37 mg/dL), indicates the prepared kit's robustness and clinical applicability. These results validate the potential of PLPOD plant-based peroxidase enzyme as cost-effective alternatives in diagnostic reagent systems particularly for use in resource-limited settings. Furthermore, the use of locally sourced PLPOD aligns with goals of sustainable biotechnology and indigenous diagnostic manufacturing, as supported by recent development studies. This kit formulation allows sensitive and accurate quantification of glucose in serum or plasma, and the substitution of plant-derived peroxidase in place of horseradish peroxidase (HRP) offers a cost-effective and sustainable alternative, especially for local diagnostic manufacturing.

## 5 Conclusion

The present study successfully purified and characterized a peroxidase enzyme from potato leaves (PLPOD) and demonstrated its effective incorporation into a glucose diagnostic kit. The enzyme displayed favorable catalytic properties, stability across physiological pH, and responsiveness to metal ions and inhibitors consistent with known plant peroxidases. The glucose diagnostic kit formulated with PLPOD showed reliable and accurate performance comparable to a standard commercial kit across both normal and diabetic serum samples. These findings support the feasibility of using plant-derived peroxidases as sustainable, low-cost alternatives for diagnostic applications. The study highlights the potential for local production of diagnostic reagents contributing to more accessible and eco-friendly healthcare solutions.

## CRediT authorship contribution statement

**Hassan M.M. Masoud:** Formal analysis, Methodology, Supervision, Writing – original draft, Writing – review & editing. **Mohammed M. Abdel-Monsef:** Writing – original draft, Resources, Methodology. **Mohamed S. Helmy:** Writing – review & editing, Software, Data curation. **Sayed S. Esa:** Software, Methodology, Formal analysis. **Doaa A. Darwish:** Writing – review & editing, Supervision, Resources, Methodology.

## Declaration of competing interest

The authors declare that they have no known competing financial interests or personal relationships that could have appeared to influence the work reported in this paper.

## Data Availability

All data and materials are available.
